# Iloprost Duration for Digital Ulcers in Systemic Sclerosis: French Retrospective Study at Two Centers and Literature Review

**DOI:** 10.3389/fmed.2022.878970

**Published:** 2022-07-06

**Authors:** Céline Jamart, Hervé Levesque, Sara Thietart, Olivier Fain, Sébastien Rivière, Ygal Benhamou, Arsène Mekinian

**Affiliations:** ^1^Service de Médecine Interne, Hôpital Rouen, Université de Rouen, Rouen, France; ^2^Sorbonne Université, APHP, Service de Médecine Interne-DMU i3, Hôpital Saint-Antoine, Paris, France; ^3^INSERM U938, Centre de Recherche Saint-Antoine, Paris, France

**Keywords:** systemic sclerosis, DUs, iloprost, outcome, digital ulcer

## Abstract

**Objective:**

Ischemic digital ulcers (DUs) are frequent and severe complications of systemic sclerosis (SSc). Treatment options for SSc-related digital vasculopathy are based on aggressive vasodilation, with the objective to improve blood flow in ischemic areas. Intravenous prostanoids are recommended to treat active DUs. However, the level of evidence for the duration of 5 days is low. Therefore, the aim of this study was to determine whether prolonging the infusion beyond 5 days increases the rate of healing of active DUs in SSc.

**Methods:**

This is an observational longitudinal retrospective bicenter study from 2000 to 2017. The objective was to compare the healing rate and time (defined by a healing of at least 50% of DUs) between two durations of iloprost administration: 5 days or less, or more than 5 days.

**Results:**

Forty-one patients, with a mean age of 47 ± 15 years at diagnosis and 32 (78%) females have been included. Systemic sclerosis was diffuse in 10 (24%) cases and 13 (32%) had an interstitial lung disease. A total of 243 iloprost infusions for DUs were performed: 140 infusions for 5 days or less, and 103 infusions for more than 5 days (prolonged duration). Patients with active DUs which received >5 days of iloprost had higher modified Rodnan skin scale at the time of iloprost infusion (median 33 vs. 15; *p* < 0.05), more interstitial lung disease (44 vs. 27%; *p* < 0.05), more anti-topoisomerase I antibody positivity (59 vs. 44%; *p* < 0.05), and received more previous cyclophosphamide therapy (48 vs. 19%; *p* < 0.05). While the number of active DUs before iloprost infusion was not significantly different among those who received ≤5 days and >5 days of iloprost, the time to healing after iloprost infusion significantly decreased in SSc patients who received >5 days iloprost infusion: 48 [7–392] vs. 91 [9–365] days (*p* < 0.05). The proportion of SSc patients with healed DUs tended to increase in patients with >5 days iloprost infusion (log rank = 0.06). The number of patients with complete DU healing at day 90 was significantly increased in SSc who received >5 days of iloprost: 53 (51%) vs. 52 (37%) (*p* < 0.05). In addition, the time to healing was not significantly associated with the use of calcium channel blockers, endothelin receptor antagonists or a combination of PDE-5 inhibitors.

**Conclusion:**

Prolonging duration of iloprost >5 days could improve the healing rate and the time to healing of SSc-related DUs. Prospective randomized studies are needed to confirm these data and define the optimal duration of iloprost therapy.

## HIGHLIGHTS

- Iloprost infusion during more than 5 days improves the healing rate and the time to healing, as well as the proportion of healed DUs at 3 months in SSc-related DUs.

- The value of endothelin receptor antagonists or a combination of PDE-5 inhibitors and of hemodilution during the iloprost infusion remains to be determined.

- Pursuing calcium channel blockers during the iloprost infusion is associated with increased infusion-related side effects.

## Introduction

Systemic sclerosis (SSc) is a severe connective tissue disease in which vasculopathy, autoimmunity and fibrosis are the key events. Despite the progress that has been made in early diagnosis and management of organ-based complications, many challenges still remain in the management of SSc. Therefore, DUs are still common in the course of SSc (from 24 to 58%), and are a major source of disability (1, 2). The presence of DUs has been identified as an independent risk factor of mortality (3). Management of DUs includes a local treatment, such as non-invasive debridement and occlusive dressings, and a systemic administration of calcium-channel inhibitors. A combination of iloprost infusions and phosphodiesterase 5 inhibitors are used in severe cases.

Several clinical trials evaluated the efficacy of iloprost for SSc-related DUs (4–8). In two multicenter double-blinded randomized trials, infusion of iloprost (0.5–2.0 ng/kg/min over 6 h, during 5 consecutive days) was associated with significantly greater proportion of DU healings in comparison to placebo (7, 8). In a meta-analysis of therapies for DU prevention and healing, oral prostanoids (iloprost, beraprost, cisaprost, and treprostinil) were not associated with less occurrence of new DUs, when compared with placebo (9).

Unlike thromboangiitis obliterans, where the duration of iloprost infusion is well-established, the optimal schedule of iloprost infusion in SSc has not yet been defined. Therefore, the benefit of prolonged iloprost therapy is uncertain for SSc patients (10). In addition, the value of additional drugs, such as endothelin receptor antagonists (ERAs, i.e., bosentan) and phosphodiesterase 5 inhibitors (PDE-5, i.e., sildenafil) concomitant to iloprost infusion remains to be determined.

In this retrospective study, we therefore aimed (1) to compare the time to healing between two regimens of ilosprost administration: duration greater than 5 days, or 5 days or less; (2) to assess the factors associated with the time to healing, particularly the benefit of concomitant use of endothelin receptor antagonists with phosphodiesterase 5 inhibitors.

## Patients and Methods

### Study Design

The study was a bicenter retrospective observational study carried out in two tertiary medical centers labeled as reference centers for SSc. All patients with SSc who received at least 3 consecutive days of iloprost infusion for DUs between January 2000 and January 2017 were included. Patients were also subjected to local therapy as debridement and topical medications.

### Patients

**FIGURE 1:**
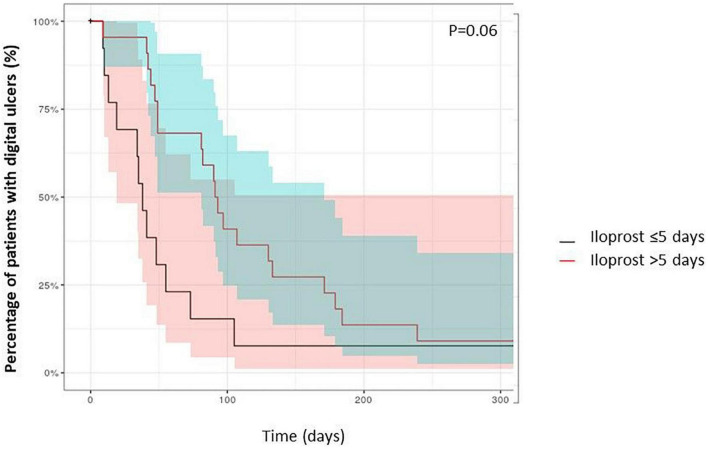
Time to healing of digital ulcers of SSc patients (Kaplan Meyer curves).

The inclusion criteria were as follows: (1) age ≥ 18 years; (2) limited or diffuse SSc (ACR/EULAR criteria) (11); (3) at least one active digital ischemic ulcer, defined as a lesion with visually discernable depth and a loss of continuity of epithelial coverage, which could be denuded or covered by a scab or necrotic tissue, localized at distal to the proximal interphalangeal joints and without bone infection or calcinosis (12); (4) at least 3 consecutive days of iloprost infusion (Figure 1).

### Data Collection

Data were collected retrospectively from patients’ charts at the time of iloprost infusion, and at each visit and/or hospitalization after the infusion during the next 90 days and until the last available visit. Baseline data, including patients’ demographics, history and presentation of SSc, organ involvement, autoantibodies, factors associated with digital ischemia (hypertension, smoking, body mass index, diabetes mellitus, cholesterol levels), and treatments were analyzed. Features of iloprost regimen were analyzed as follows: infusion rate, peripheral or central intravenous line, duration and tolerance. Each iloprost cession was considered and all iloprost cessions were pooled to analyze a cumulative prevalence of ulcers healing and time to healing. Healing was defined as complete re-epithelialisation and was considered only when it was notified in the medical files.

### Ethical Considerations

This study was conducted in compliance with the Good Clinical Practices protocol and Declaration of Helsinki principles. The study being retrospective and observational, French law did not require formal approval from an ethics committee.

### Statistical Analyses

Descriptive statistics [mean, median, range, standard deviation (SD)] are reported for quantitative variables. Numbers and percentages were calculated for categorical variables. To identify potential confounding factors influencing DU healing, we used Fisher’s exact test or Mann Whitney test, and univariate regression analyses were performed. We analyzed a pooled analysis of all active ulcers among 41 patients which received at least one iloprost infusion (a total of *n* = 243 infusions), and compared patients which received ≤5 days and >5 days of iloprost infusions and determined the time to healing. The Gray cumulative model was used to consider the comparison between all pooled patients iloprost lines of therapy depending on the number of days of iloprost infusion. A *p* value less than 0.05 was considered as significant.

## Results

### Patients’ Characteristics

Forty-one patients (32 females, mean age 47 ± 15 years) had been included. Disease duration from the first non-Raynaud symptom was 6 ± 5.2 years. SSc was diffuse in 10 (24%) cases and 13 (32%) have an interstitial lung disease (Table 1). Prevalence of anti-topoisomerase I, anti-centromeres and anti RNA polymerase III antibodies was 16 (40%), 12 (30%), and 3 (7.5%), respectively.

**TABLE 1:** Characteristics of 41 patients with SSc.

Characteristics	*N* (%)
**Demographics**	
Number of patients	41
Female, *n* (%)	32 (78)
Age at inclusion, yrs, mean ± SD	47 ± 14.7
Caucasian, *n* (%)	27 (66)
Sub-Saharan Africa, *n* (%)	10 (24)
**Clinical characteristics**	
Diffuse SSc, *n* (%)	10 (24,4)
Limited SSc, *n* (%)	31 (76)
Time since first Raynaud Phenomenon, yrs, mean ± SD	8.7 ± 8.2
Time since first non-Raynaud Phenomenon symptom, yrs, mean ± SD	6 ± 5,18
Time since first ischemic DU, yrs, mean ± SD	3.5 ± 4.5
**Organ involvement**	
Pulmonary arterial hypertension, *n* (%)	2 (5)
Interstitial lung disease, *n* (%)	13 (32)
History of renal crisis, *n* (%)	0 (0)
Esophagus involvement, *n* (%)	28 (68)
Chronic intestinal pseudo-obstruction, *n* (%)	1 (2)
**Immunological characteristics**	
Antinuclear antibodies, *n* (%)	39 (95)
Anti-topoisomerase 1 antibodies, *n* (%)	16 (40)
Anti-centromere antibodies, *n* (%)	12 (30)
Anti RNA polymerase III antibodies, *n* (%)	3 (7.5)
**Cardiovascular risk factors**	
No history of smoking, *n* (%)	26 (63)
Active smoking, *n* (%)	13 (32)
Diabetes mellitus, *n* (%)	2 (5)
Hypertension, *n* (%)	2 (5)
Hypercholesterolemia, *n* (%)	2 (5)
Overweight (BMI > 25), *n* (%)	15 (38)

*SSc, systemic sclerosis; UD, digital ulcer.*

### Iloprost Infusions for Digital Ulcers

**FIGURE 2:**
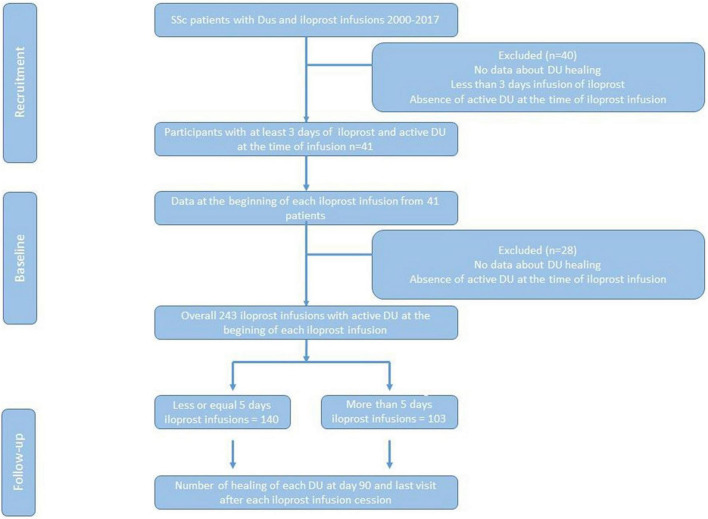
Study flow chart.

**FIGURE 3:**
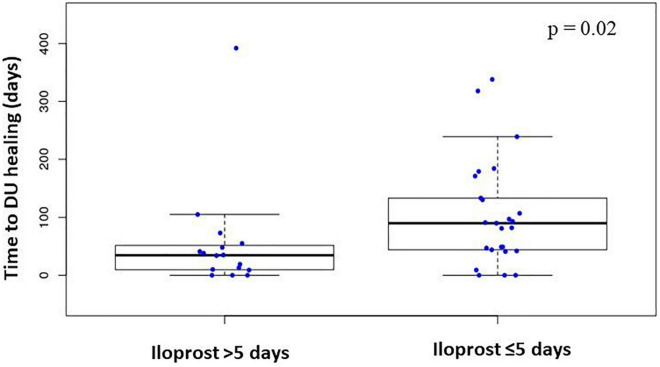
Time to digital ulcers healing in SSc patients with iloprost infusion less or more than 5 days.

Overall, 41 patients received 243 iloprost infusions for DUs. The median infusion duration was at 7.3 days (3–28), with a median infusion rate of 1.7 ng/kg/min. The characteristics between patients who received ≤5 days of iloprost (*n* = 140 infusions) to those who received >5 days iloprost therapy (*n* = 103 infusions) are detailed in Table 2, and all patients received consecutive iloprost infusions. The median number of iloprost cessions was 3 (1–12) with median interval between iloprost cessions at 6 weeks (4–42). The number of active DU before overall iloprost infusions was at 3 (1–12). Patients received immunosuppressive therapies at the time of iloprost infusion in 123 cases, with cyclophosphamide (*n* = 101), mycophenolate mofetil (*n* = 8), methotrexate (*n* = 47), and rituximab (*n* = 14). Patients with active DUs which received iloprost during >5 days had a higher modified Rodnan skin scale at the time of iloprost infusion (median 33 vs. 15; *p* < 0.05), more frequent interstitial lung diseases (44 vs. 27%; *p* < 0.05), anti-topoisomerase I antibodies positivity (59 vs. 44%; *p* < 0.05), and previous cyclophosphamide therapy (48 vs. 19%; *p* < 0.05) (Table 2). Healing time after iloprost infusion was significantly lower in SSc patients who received >5 days of iloprost [48 (7–392) days] in comparison with patients with ≤5 days of iloprost infusion [91 (9–365) days; *p* < 0.05] (Figure 2). The number of active DUs was similar between the two groups: 3 (1–12) vs. 3 (1–10) (*p* = 0.2). There was a tendency toward a higher proportion of patients with healed DUs among patients with a longer iloprost regimen (log rank = 0.06) (Figure 3). The number of patients with complete DU healing at day 90 was significantly higher among patients who received >5 days of iloprost: 51 vs. 37% (*p* < 0.05).

**TABLE 2:** Systemic sclerosis features and DUs outcome in patients treated with ≤5 days and >5 days of iloprost infusions: data expressed as number of all lines of iloprost infusions from 41 SSc patients.

Characteristics	Iloprost duration ≤ 5 days *N* = 140 lines of iloprost infusions from 41 patients	Iloprost duration > 5 days *N* = 103 lines of iloprost infusions from 41 patients
**Demographics**		
Age at diagnosis, medians (ranges)	45 (17–83)	47 (17–78)*
Caucasian, *n* (%)	66 (47)	47 (46)
Sub-Saharan Africa, *n* (%)	45 (32)	43 (42)
**Clinical characteristics**		
Limited SSc, *n* (%)	85 (61)	69 (67)
Time since first non-RP symptom, median (ranges)	7 (0–21)	10 (0.2–20)
**Organ involvement**		
Pulmonary hypertension, *n* (%)	13 (9)	12 (12)
Interstitial lung disease, *n* (%)	38 (27)	45 (44)*
Heart, *n* (%)	9 (6)	7 (7)
GERD, *n* (%)	120 (86)	83 (81)
Joint, *n* (%)	76 (54)	49 (48)
**Immunological characteristics**		
Anti-topoisomerase 1 antibodies, *n* (%)	62 (44)	61 (59)*
Anti-centromere antibodies, *n* (%)	19 (14)	13 (13)
Anti-ARN polymerase III antibodies, *n* (%)	25 (18)	13 (13)
**Cardiovascular risk factors**		
Diabetes mellitus, *n* (%)	9 (6)	6 (6)
Hypertension, *n* (%)	36 (26)	22 (21)
Overweight (BMI > 25), *n* (%)	45 (32)	25 (24)
**Previous immunosuppressive therapies**		
Cyclophosphamide, *n* (%)	26 (19)	49 (48)*
Mycophenolate mofetil, *n* (%)	5 (4)	3 (3)
Methotrexate, *n* (%)	25 (18)	22 (21)
Rituximab, *n* (%)	9 (6)	5 (5)
Modified Rodnan skin scale before infusion, medians (ranges)	15 (1–36)	33 (1–37)*
Number of DUs before infusion, medians (ranges)	3 (1–12)	3 (1–10)
**Treatment during iloprost infusion**		
Calcium channel blockers, *n* (%)	91 (65)	51 (49)*
Phosphodiesterase 5 inhibitors, *n* (%)	35 (25)	14 (14)*
Endothelin receptor antagonists, *n* (%)	6 (4)	14 (14)*
Hemodilution, *n* (%)	31 (22)	19 (18)
Healing time (days), medians (ranges)	91 (9–365)	48 (7–392)*
Healing at day 90, *n* (%)	52 (37)	53 (51)*
Infection, *n* (%)	11 (8)	9 (9)

GERD, gastroesophageal reflux disease; *p < 0.05.

Use of calcium channel blockers and PDE-5 inhibitors was less frequent among patients who received >5 days of iloprost, in comparison with patients with the shorter regimen, whereas the concomitant use of endothelin receptor antagonists was more common in these patients (Table 2). Healing time was not significantly modified by the use of calcium channel blockers, endothelin receptor antagonists or of a combination of PDE-5 inhibitors. In addition, the number of DUs and the type of immunosuppressive therapies did not influence healing (data not shown).

The number of DU complications (osteitis and/or gangrene and/or amputation) was not significantly different between groups (*p* = 0.42).

### Safety

At least one adverse event was noted in 51% cases (mostly headaches and nausea), and 20% presented severe side effects (hypotension, incoercible vomiting). Interestingly, adverse events were more common in patients who received concomitant calcium channel blockers (63 vs. 56%; *p* = 0.045).

## Discussion

The determination of the optimal duration of iloprost infusions remains a challenge. We report in this retrospective study that extending iloprost treatment for more than 5 days could be an attractive option for active DUs, especially in patients with severe diffuse SSc. Indeed, prolonged infusions of iloprost shortened the healing time by half, in comparison with patients with shorter treatment duration. The value of endothelin receptor antagonists or the combination of PDE-5 inhibitors remains to be determined, while continuation of calcium-channel blockers was associated with higher proportion of adverse side effects.

Although there is some evidence from clinical trials to use iloprost for SSc-related DUs, administration of iloprost in clinical practice follows various non-validated regimens, often based on the physician’s experience (Tables 3, 4; 2–8, 10–28). In addition, some reports have pointed out that long-term recurrent administration of iloprost was associated with no recurrence of DU (20). Unlike thromboangeitis obliterans or peripheral artery disease, the optimal duration and the frequency of iloprost infusions in SSc patients are uncertain. Therefore, guidelines do not provide any recommendations on iloprost dosage and regimen. Herein, using a clinical endpoint (the healing of DUs), the use of a prolonged regimen of iloprost was associated with a shorter healing time and a trend toward higher proportions of patients with healed DUs.

**TABLE 3:** Literature review of randomized trials using iloprost for DUs in systemic sclerosis.

Authors/Years	N/n with DUs	Treatments	Follow-up	Efficacy
Rademaker et al. (5)	23/-	Iloprost ≤ 2 ng/kg/min ×8 H/d ×3 days + 1 infusion at W8 vs. nifedipine 30 mg/d (4 weeks)	16 weeks	Number of DUs from 3.5 ± 1.6 to 0.6 ± 0.3 under iloprost vs. 4.3 ± 0,8 to 1.4 ± 0.5 under nifedipine [11]
McHugh et al. (4)	29/12	Iloprost ≤ 2 ng/kg/min ×6 H/d ×3 days vs. placebo, cross over at 6 weeks	2 × 6 weeks	No difference vs. placebo
Wigley et al. (7)	35/11	Iloprost 0,5–2 ng/kg/min ×6 H/d × 5 days vs. placebo	10 weeks	Healing of DUs: 4/4 under iloprost vs. 0/4 under placebo (*p* = 0.029)
Wigley et al. (8)	131/73	Iloprost 0, 34–1.91 ng/kg/min ×6 H/d ×5 days vs. placebo	9 weeks	Decrease > 50% of number of DUs: at 3 weeks: 20% under iloprost vs. 5.4% under placebo (*p* = 0.06) at 6 weeks: 28.1 vs. 15.2% [11] at 9 weeks: 25.7 vs. 18.4% [11] Decrease of 36% of DUs under iloprost vs. 14.1% under placebo (*p* = 0.064)
Torley et al. (6)	55/15	Iloprost 0,5 ng/kg/min vs. 2 ng/kg/min ×6 H/d × 3 days	8 weeks	At W0: 23 DUs under placebo vs. 16 under iloprost, At W8: 14 DUs under placebo vs. 9 d under iloprost, Decrease of 39% under placebo vs. 44% under iloprost [11]
Scorza et al. (28)	46/17	Iloprost: ≤2 ng/kg/min ×8 H ×5 d than 1 infusion/6 weeks vs. nifedipine 40 mg/d	12 months	DUs healing in 3/3 patients under nifedipine and 12/14 patients under iloprost [11]

**TABLE 4:** Literature review of observational non-randomized trials using iloprost for DUs in systemic sclerosis.

Authors/Years	Type of study	N/n with DUs	Iloprost regimen	Follow-up	Efficacy
Biasi et al. (15)	Prospective observational	20/-	Iloprost 0.5–2 ng/kg/min ×6 h/d ×5 days/month for 1 year	12 months	Number of digital ulcers from 31.8 ± 19.1 to 2.2 ± 2.0 (*p* < 0.05)
Bettoni et al. (14)	Prospective observational	30/21	Iloprost 0.5–2 ng/kg/min ×6 h/d ×5 days and 1 day/3 weeks for 3 years	Median of 3 years	Complete healing in 90% [11]
Airo et al. (13)	Retrospective exposed/not-exposed	54/47	Iloprost 0.5–2 ng/kg/min ×6 h/d ×5 days puis 1 day/3 weeks for 48 months	Median of 48 months (17–108)	Complete healing in 29 patients (62%) [11]
Scarsi et al. (25)	Retrospective	59/50	Iloprost 0.5–2 ng/kg/min ×6 h/d ×5 days and 1 day/3 weeks for 52 months	Median of 52 months	Complete healing in 35 patients (70%) [11]
Caramaschi et al. (18)	Retrospective	85/29	Iloprost 0.5–2 ng/kg/min ×6 h/d for 1 day/months	Median of 86 months	37.9% of DUs before iloprost vs. 20.7% after iloprost [11]
Caramaschi et al. (17)	Retrospective	115/41	Iloprost 0.5–2 ng/kg/min ×6 h/for 1 days/month	98.8 ± 37.5 months	0.31 imputation per 100 patients-year under iloprost
Colaci et al. (19)	Retrospective	55/31	Iloprost 0.8–1 ng/kg/min ×6 H/for 3 days	10 ± 4.2 years	Complete healing in 71% cases
Foti et al. (20)	Retrospective	68/29	Iloprost 0.5–2 ng/kg/min ×;6 h/d for 6 days/months	7.1 ± 2.9 years	42.6% DUs before iloprost vs. 11.8% after iloprost (*p* < 0.001)

Systemic sclerosis microvascular structural damage and dysfunction represent the initial morphological and functional markers of the disease. Some authors have reported that patients receiving iloprost had an improvement on Doppler and videocapillaroscopy characteristics, when compared with patients without iloprost (29). Unfortunately, these vascular effects were no longer seen at the next infusion (26). Several cappilarscopic and ultrasonographic tools could help to the clinical evaluation of DU healing, and Ultrasound classification of finger pulp blood flow was correlated with the risk of DU (30, 31). We were unable to obtain the results of the vascular examinations, which could have strengthened our data on the benefit of prolonged use of iloprost. However, the beneficial effects of prolonged infusion of iloprost is supported by the mechanisms of the drug. Prostanoids are potent vasodilators, and inhibit platelet aggregation and vascular smooth muscle cell proliferation. In SSc, DUs involve both microvessels and digital arteries (20). Moreover, an increased prevalence of macrovascular diseases proximal to the digital artery has been reported in SSc (27), in particular affecting the ulnar artery. After iloprost, a significant improvement in endothelial-dependent vasodilation was seen only in SSc patients with an “active” Nailfold videocapillaroscopy pattern. The iloprost effects vanished within 7 days after the last infusion (32). Therefore, our results are consistent with those of thromboangeitis obliterans, where a prolonged duration of infusion is required (10, 29). Patients under iloprost have Doppler and videocapillaroscpy improvement in comparison with those without iloprost, but these vascular effects were no longer observed at the following infusion (26).

The other major result is the benefit of concomitant vasoactive drugs. Two extensive, multicenter placebo-controlled studies have proved bosentan to be an effective treatment option in preventing new DUs and in the treatment of current DUs in relatively small series (22, 23). However, the beneficial effect of combining bosentan with iloprost has not yet been reported. Here, we found no improvement with this dual therapy, particularly among patients with the longer iloprost regimen, as they received more frequently an endothelin receptor antagonist. This result is consistent with the results of RAPIDS-2 (for the Randomized, double-blind, Placebo-controlled study with bosentan on the healing and prevention of Ischemic Digital ulcers in patients with systemic Sclerosis), where treatment with bosentan reduced the occurrence of new DU in patients with SSc, but had no effect on healing of DU compared to placebo (23). To date, it is unclear why a drug that presumably promotes vasodilation as an antagonist of ET-1 would not also promote ulcer healing. One hypothesis is that the expression of endothelin receptors on keratinocytes suggests that ET-1 could be a modulator of function and, therefore, receptor blockade might impair epithelialization despite beneficial effects on other aspects of healing. Likewise, PDE-5 inhibitors have also been studied for curing DU in SSc. In a French randomized study, even though the healing time was not improved under sildenafil, there was a significant decrease in the number of DUs in favor of sildenafil at the 8th and 12th week (33). To our knowledge, data is scarce on this aspect, and we have not found data suggesting that these various drug combinations could improve healing time in patients with SSc. However, the present study could not allow us to draw a definitive conclusion, due to the small number of patients treated with each drug and combination of drugs.

Prolonged duration of iloprost can be a source of morbidity and disability, due to increased frequency and duration of hospitalizations and venous access. In addition, no criteria has yet been determined to stop the treatment. It is questionable to prescribe a drug that can possibly be responsible for side effects, without clear guidelines based on robust studies. Another important problem relates to strategies for preventing the recurrence of DUs, and in particular the preventive use of endothelin receptor antagonists or PDE-5 inhibitors. Only a small proportion of our cohort was treated by endothelin receptor inhibitor, though this drug is known as an effective preventive drug for DU recurrence (22, 23), even though 90% of the infusions were performed for recurrent DUs. However, these data are similar to a recent analysis from the observational real-life DESScipher study, showing that the proportion of patients with a combination therapy is still low with 32.6% patients under 2 drugs and 11.5% under three or more therapies (16).

Several limitations should be addressed, in particular the retrospective nature of the study and the limited number of iloprost infusions. In addition, the classification criteria for defining healing are heterogeneous. Finally, the duration of iloprost infusion has been based on the physician’s experience and may therefore differ between two medical centers. Despite these limitations, a prolonged use of iloprost in patients with severe diffuse SSc with dominant microcirculatory involvement is coherent and seem to improve the healing rates.

To conclude, a prolonged administration of iloprost may improve the healing rate and the time to healing of DUs related to SSc. Prospective randomized studies are guaranteed to confirm these data and define the optimal duration of iloprost.

## Data Availability Statement

The original contributions presented in this study are included in the article/supplementary material, further inquiries can be directed to the corresponding author.

## Author Contributions

CJ, HL, OF, SR, YB, and AM: conception and design of the study and analyzing and interpretation of data. CJ and AM: acquisition of data. CJ, HL, ST, OF, SR, YB, and AM: drafting and final approval of the manuscript. All authors contributed to the article and approved the submitted version.

## Conflict of Interest

AM was investigator of CELGENE, ROCHE, CHUGAI founded trials with APHP and Hôpital 15-20 promotion; AM received several fees for congress travels and experts’ use from LFB, SANOFI, SHIRE, and CELGENE. The remaining authors declare that the research was conducted in the absence of any commercial or financial relationships that could be construed as a potential conflict of interest.

## Publisher’s Note

All claims expressed in this article are solely those of the authors and do not necessarily represent those of their affiliated organizations, or those of the publisher, the editors and the reviewers. Any product that may be evaluated in this article, or claim that may be made by its manufacturer, is not guaranteed or endorsed by the publisher.
